# Maternal Near-Miss Secondary to Concealed History: A Case Report

**DOI:** 10.7759/cureus.39473

**Published:** 2023-05-25

**Authors:** Shivangni Sinha, Mukta Agarwal, Smita Singh, Simran Dureja

**Affiliations:** 1 Obstetrics and Gynaecology, All India Institute of Medical Sciences, Patna, Patna, IND

**Keywords:** obstetrical cause, maternal mortality, multiple organ dysfunction syndrome, severe acute maternal morbidity, maternal near miss

## Abstract

A mother and her child constitute an essential part of the healthcare system. Maternal death due to obstetrical causes is tragic for the family and the healthcare system. A maternal near-miss is a woman who survived problems during pregnancy and childbirth and has been examined as an intermediary for maternal deaths. Reviews of such situations are viewed as a less risky strategy by the service provider to improve maternal health care. This will allow us to take advantage of possibilities to prevent the deaths of mothers who may meet a similar fate. This is the case of a survivor of pregnancy termination challenges whose concealed history eventually led to a series of events compromising her health to a near-mortality condition. Providing complete information to a clinician is a crucial component of quality healthcare, as a family is the first in contact with a patient. The significance is evident in this case report.

## Introduction

Pregnancy and childbirth difficulties can occur at any time, so healthcare centers need to be sufficiently prepared for the timely management of complications and their consequences. Any pregnant woman in a serious state requires immediate emergency care. Identifying these danger signs with family and clinicians is important [1].

A near-miss is a pregnant or recently delivered woman who was critically ill but survived a problem during pregnancy, childbirth, or within 42 days of termination of pregnancy. Severe acute maternal morbidity (SAMM) is a term used to describe life-threatening illnesses that can result in a near-miss with or without residual morbidity or fatality. Women who experienced SAMM during pregnancy have numerous clinical and environmental variables in common with those associated with maternal mortality. Severe maternal outcome (SMO) refers to the combination of near-miss cases and maternal deaths [1]. Thus, the concept of near-miss was established to supplement concepts gathered from maternal mortality reviews [2]. 15.6% of cases of near-miss are provoked by a pre-existing disease that, when overlooked, succumbs to mortal outcomes [2].

We provide a case report of a patient who concealed her past, yet correct clinical evaluation, quick decision, timely intervention, and prudent management rescued her from inadvertent death. Written informed consent was obtained for publication.

## Case presentation

Case report 1

A 22-year-old patient reported to our emergency department in a tertiary care center complaining of stomach fullness, respiratory distress, and difficulties passing urine for the past 24 hours. The patient was multiparous, with a history of previous 2LSCS (4 years and 2 years, respectively). She gave a history of undergoing suction evacuation 24 hours ago for a period of gestation (POG) at a private local nursing home. No documentation was available, and the patient and family failed to provide any other information. According to her, she began complaining of abdominal pain immediately following the treatment, which was increasing in nature. The patient was given some pain-relieving drugs. The pain worsened over time, and after six hours, she suffered respiratory distress and abdominal fullness, prompting her family to seek medical attention at our emergency department.

On physical examination, she was severely pale with respiratory discomfort while supine. Her blood pressure was recorded at 100/60bpm, and her pulse at 140/min. Chest auscultation showed bilateral coarse crepitation in basal areas. On abdominal examination, generalized guarding and fullness were present, with a dull note on percussion and an absent bowel sound on auscultation. Primary investigations were sent; blood/ICU/ventilator was arranged. Following detailed counseling and with the consent of "death on the table", the patient was immediately taken up for an emergency laparotomy. She was diagnosed with severe metabolic acidosis with DIC, acute renal injury, and with acute respiratory insufficiency (MODS) as per the WHO criteria for severe maternal morbidity [3] (Table 1). 

**Table 1:** Baseline involvement of organ systems with respect to the WHO criteria [3].

System involved	Specific criteria (WHO criteria for severe maternal morbidity)	Our patient
Cardio-vascular system	Shock, cardiac arrest, pH < 7.1, Lactate >5 mmol/L, 45 mg/dL CPR, vasoactive drugs	pH <7.02, shock +ve Lactate 7.9 mmol/L D-dimer: 5.63
Respiratory system	Acute cyanosis, gasping, severe tachypnea, severe bradypnoea PAO2/FiO2 <200, O2 saturation< 90% for >60 min Intubation and ventilation not related to anesthesia	Chest: B/L coarse crepitation SpO2: 62% on room air Intubated on ventilator (CPAP) maintaining FiO2 of 60% PEEP: 9cm H2O
Renal system	Oliguria non-responsive to fluids or diuretics, Creatinine> 300 µmol/ml or 3.5 mg/dL Dialysis for acute renal failure	Urine output: 305ml in 24hrs Creatinine: increasing trend from 4.57 to 6.31mg/dl 5 cycles of dialysis
Coagulation	Failure to form clots Platelets < 50,000/ml massive transfusion of blood or red cells (>/= 5 units)	aPTT = 42.8 PT/INR: 18.2/ 1.35 Massive transfusion: 5pint PRBC, 4pint FFP, 4pint Platelet
Hepatic	Jaundice in the presence of preeclampsia Bilirubin> 100 or 6 mg/dL	Direct/Total Bilirubin: 4.12/6.15 SGPT/OT: 29/115 mg/dL
Neurological	Prolonged unconsciousness/coma (lasting > 12 h) stroke, status epilepticus/uncontrollable fits or global paralysis	-------------
Alternative severity proxy	Hemorrhage or infection leading to hysterectomy	Hysterectomy and bladder repair done

**Figure 1:**
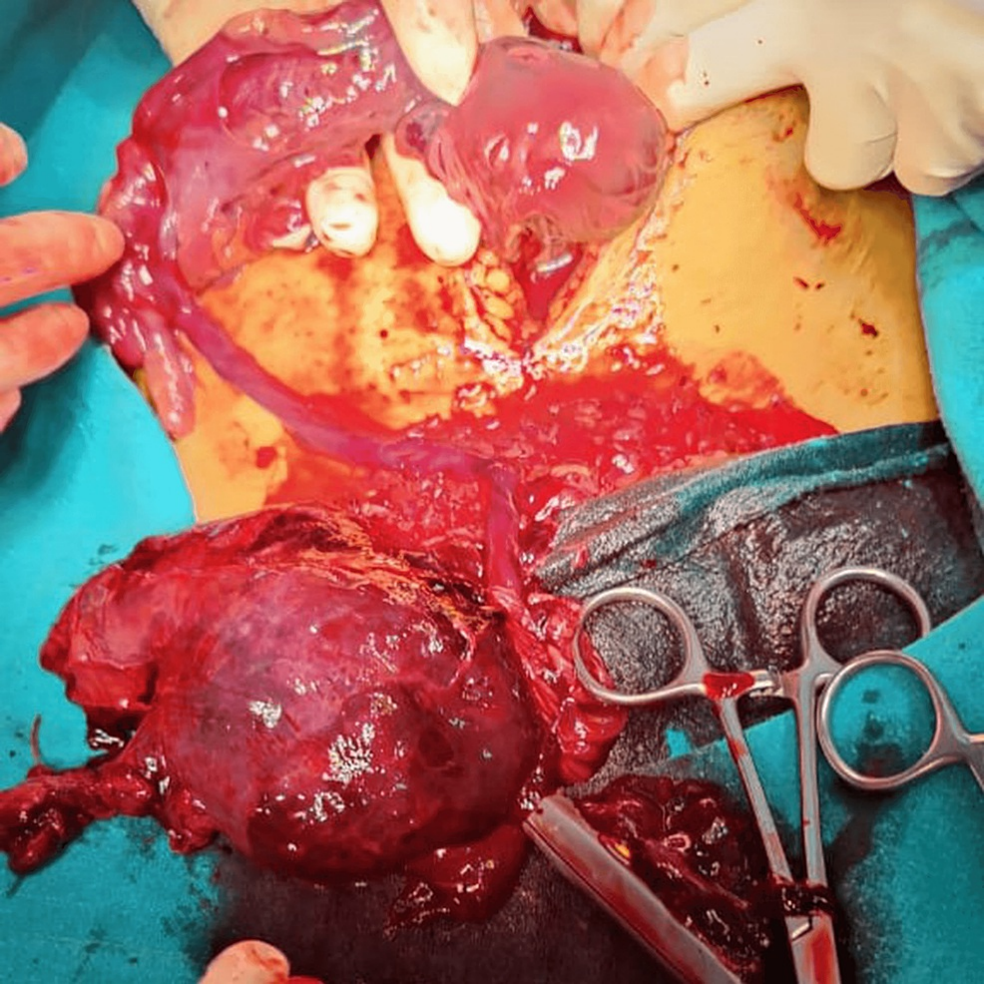
Intra-operative picture illustrates macerated fetus with placenta present intra-peritoneum after uterine rupture

**Figure 2:**
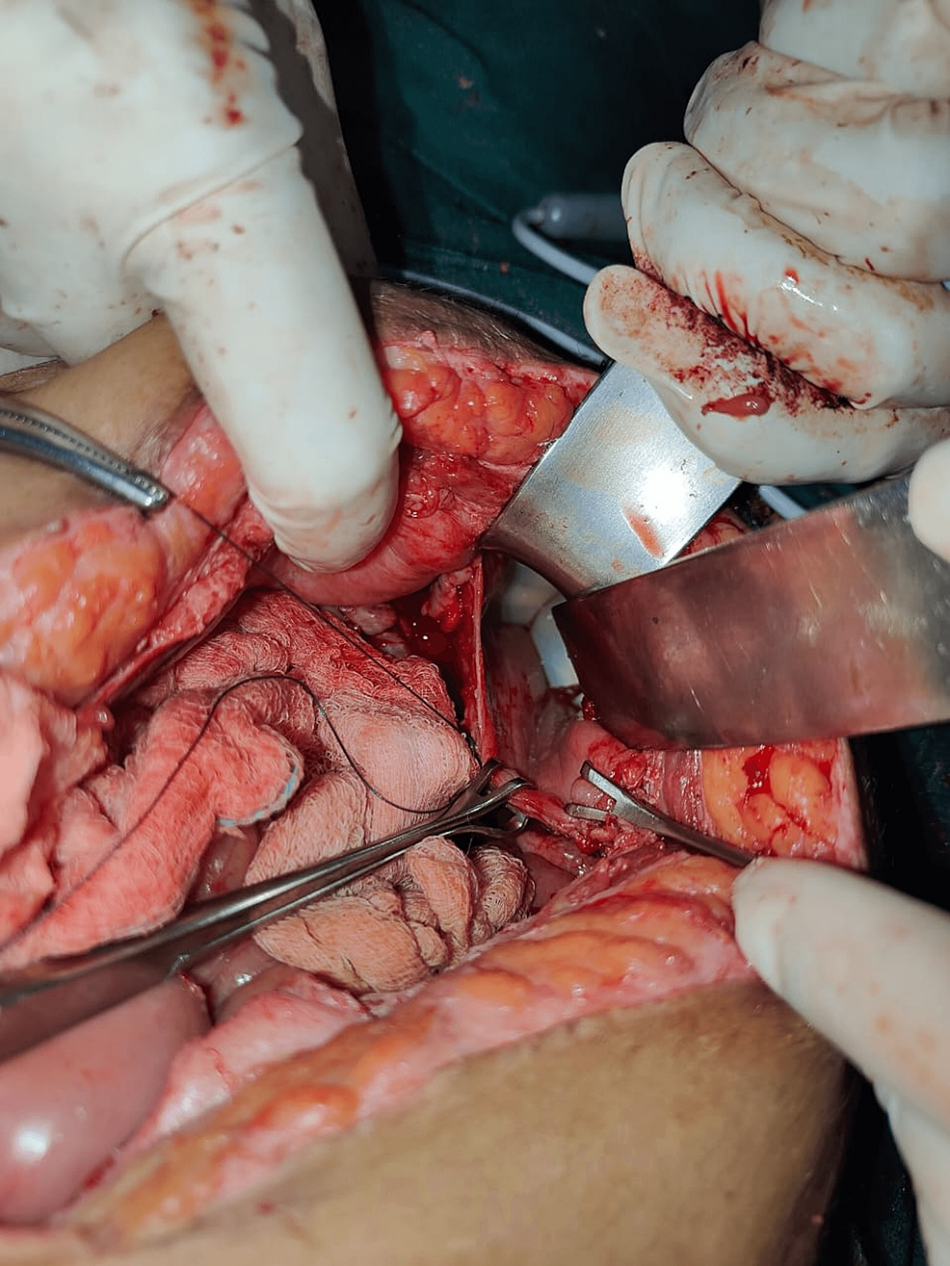
Intra-operative picture illustrating injured bladder with Foley bulb visible

**Figure 3:**
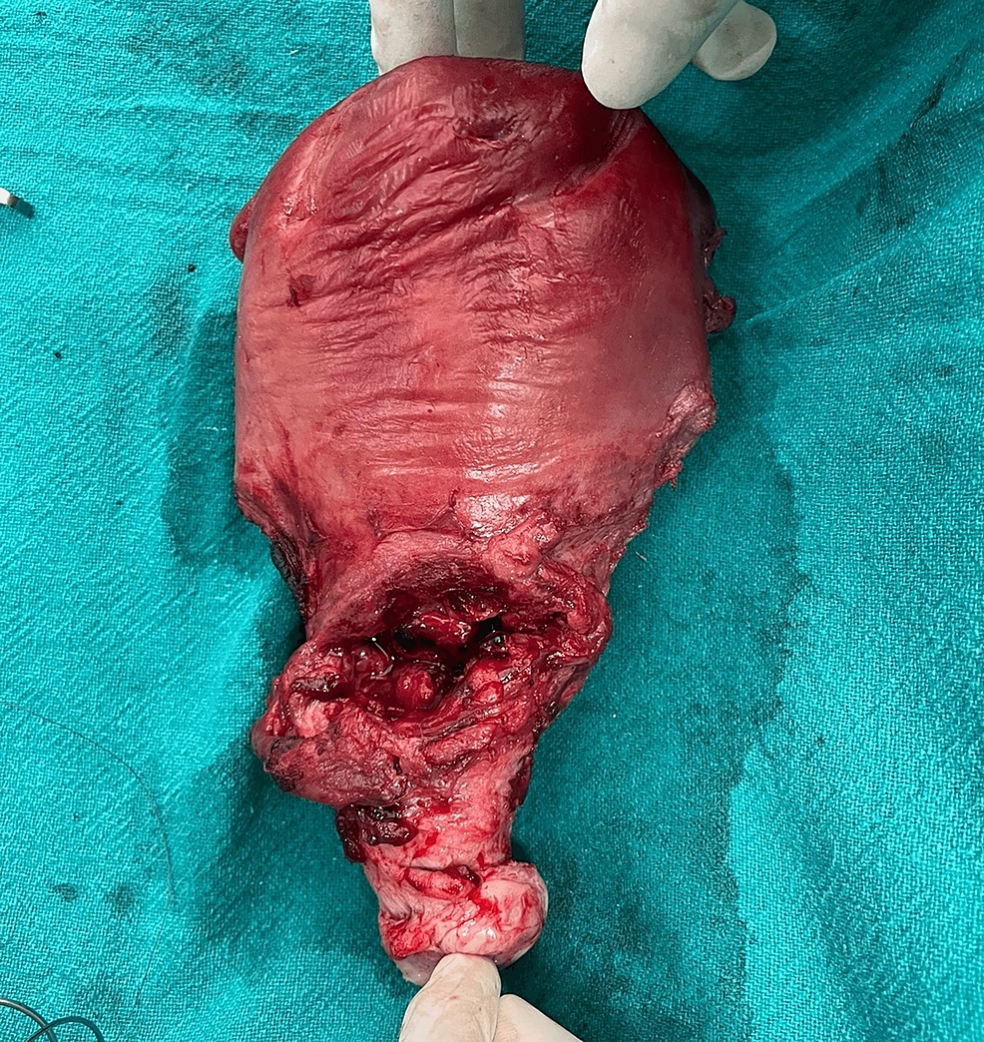
Uterine rupture at the site of a previous caesarian scar

**Figure 4:**
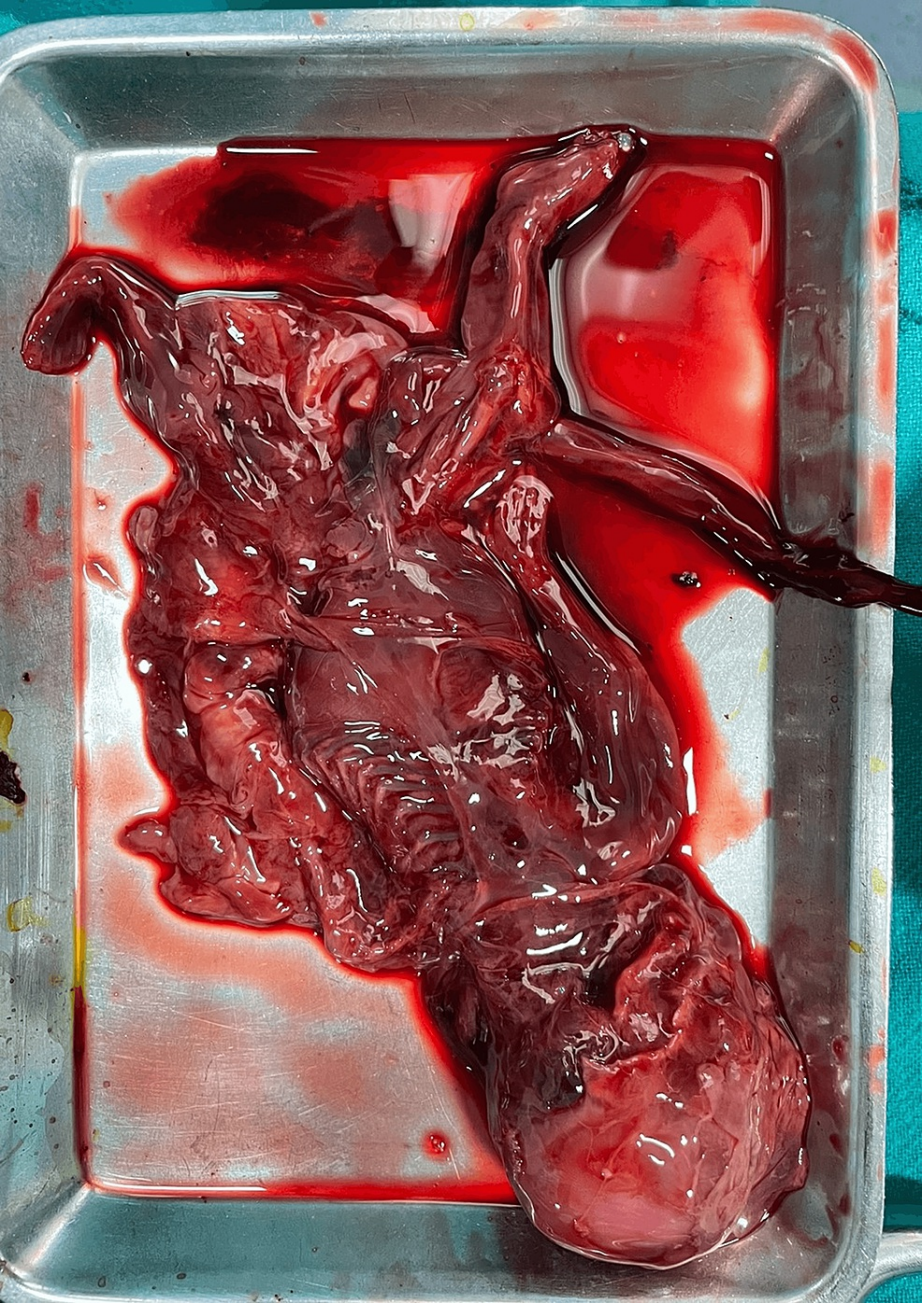
Macerated fetus corresponding to 22-24 weeks of gestation

Intraoperative findings revealed the history that was concealed in terms of the probable gestational age and the series of events that took place before and after the suction evacuation, resulting in such a fatality. There was an anterior wall uterine rupture with a rupture of the dome of the bladder. The fetus (foul-smelling) with placenta was floating in the abdomen, corresponding to six months of gestation. She underwent a hysterectomy with bladder repair (Figures 1-4).

**Figure 5:**
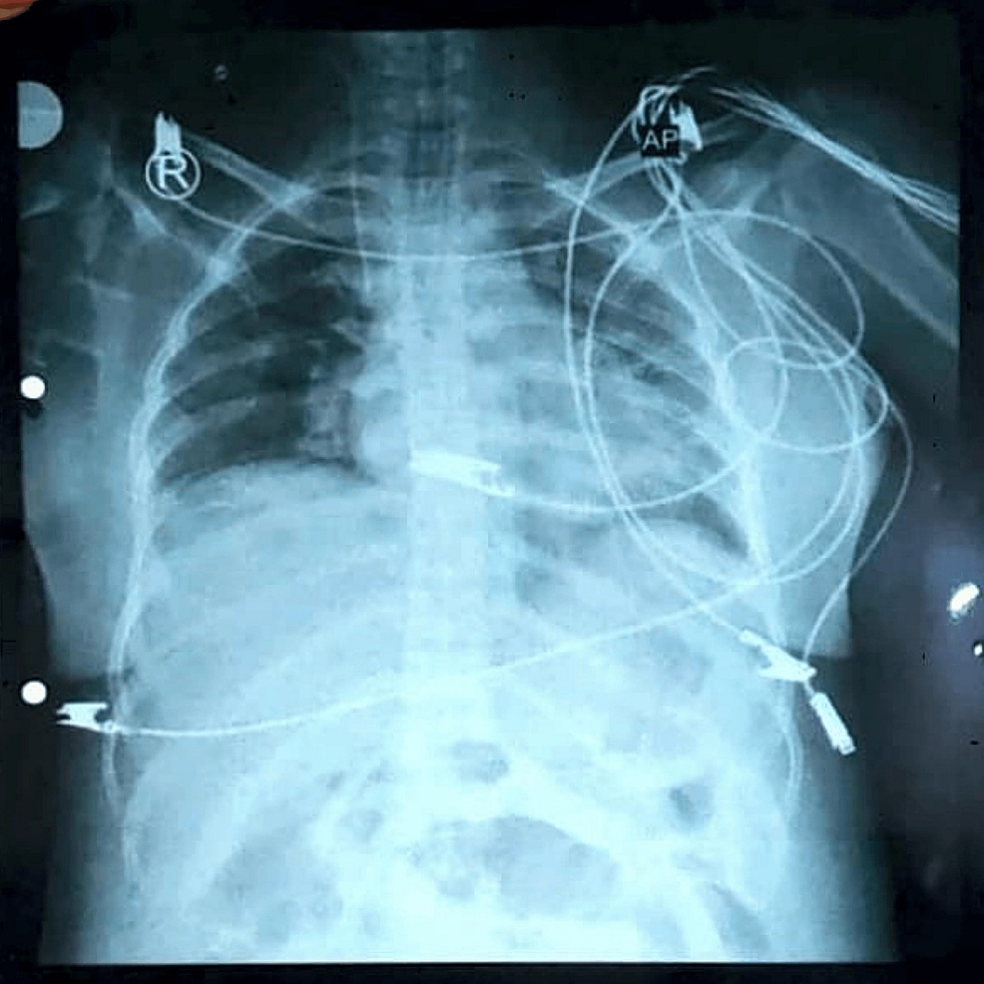
A chest X-ray of the patient shows pronounced hypoaeration, bilateral granular opacities in the pulmonary parenchyma, and peripherally extending air bronchograms suggestive of acute respiratory distress syndrome

Her postoperative course was intensive; she stayed intubated for 10 days on inotropes. There was respiratory insufficiency (Figure 5).

She was treated under a massive transfusion protocol. Her renal functions were severely compromised, leading to 5 cycles of hemodialysis on postoperative days 1, 3, 5, 6, and 8. She later developed a high-grade fever. Her USG showed raised echogenicity in the bilateral renal parenchyma, likely residual disease with mild ascites. The patient was started on injections of Meropenem and metronidazole, followed by injections of Aztreonam, clindamycin, and Linezolid as per the culture sensitivity of the endotracheal aspirate. The patient stayed febrile, showing little signs of improvement. Meanwhile, on day 14 postoperatively, wound dehiscence was observed, which was locally managed by daily sterile surgical dressing and injection of polymyxin and tablet cotrimoxazole as per wound swab culture sensitivity. Foley's catheter was removed on day 21, followed by secondary re-suturing on the 22nd day postoperatively after sterile wound swab culture and a sensitivity report (Table 2).

**Table 2:** Culture-sensitivity of body fluids

Culture Reports	Result
Blood Culture	Sterile
Urine Culture	Sterile
Endo-Tracheal Aspirate	Acinetobacter Baumanii
Wound Swab	Enterococcus sp.

With a multidisciplinary approach involving active chest physiotherapy, continued nebulization with Duolin and Budecort, and intensive care in the ICU and ward, she was saved and discharged on postoperative day 35 in stable condition. She was advised to have follow-ups monthly; however, the patient was non-compliant with follow-ups.

## Discussion

While a clinical cause is often blamed for maternal near-miss (MNM), most occurrences are the consequence of a cascade of events, including numerous social and cultural factors. Similarly, the patient/family plays an important role in assisting with a correct diagnosis, which will further determine the plan of therapy and reduce time wastage. Quite often, the family or patient is uninformed of the need for care, resulting in life-threatening situations. According to studies, almost 75% of high-risk pregnancies arrive at healthcare facilities in critical condition [4]. A review study on maternal near-misses in India was conducted between 2010 and 2019 and found that the first-level delay in seeking aid (under three-delay models) accounted for the majority of maternal near-misses, followed by the second and third-level delays (reaching out to the health care center and receiving quality care at the center, respectively) [5].

Severe acute maternal morbidity (SAMM) is also linked to long-term negative outcomes in numerous organ systems. A well-recognized long-term sequel to the injury was reported in a one-year prospective cohort investigation of near-miss cases. Patients with reduced future fertility following hysterectomies, Sheehan's syndrome in post-partum hemorrhage (PPH) surviving patients (2.6%), Asherman's syndrome (0.07%), and dyslipidemia as a prelude to cardiovascular disease (13.5%) were also identified [6]. SAMM displayed a postpartum depression prevalence rate of 23.68%, compared to global averages of 10-15 and 20%-25% in India [7].

MNM prevalence ranges from 3.9 to 379.5 per 1000 live births and 7.6-60.4 per 1000 deliveries [8]. Maternal deaths are regarded as "the tip of the iceberg" when it comes to maternal severe morbidity (MSM) [9]. As a result, the Government of India created the Maternal Death Review Guideline, which is a tool available at multiple levels to objectively examine health system performance. They assist in identifying potential gaps in the system that require attention to create plans to reduce maternal mortality and near-miss cases, thereby overcoming the system's shortcomings in providing quality care [10]. The use of the maternal near-miss concept will help to assess maternal health and maternal care quality because it provides significant information on severe morbid circumstances that, if not handled promptly, could result in fatal results. As a result, it provides an opportunity to learn about and enhance quality health services [10].

The case report defines the importance of the information that should be provided at a healthcare center, anticipating the fatality it might cause in the event of miscommunication. Public awareness about the danger signs and their outcomes needs to be addressed. A multidisciplinary, timely approach, both from patients and healthcare centers, are required to help manage near-miss cases.

## Conclusions

Surveillance and identification of factors contributing to maternal near-miss and maternal mortality are critical since they directly reflect the health system's quality of treatment. Preventable causes can be prudently minimized through communication and patient awareness in the early detection of danger signs. Notifying such cases and reviewing near-miss and mortality meetings every month will provide better knowledge of the changes needed for quality improvement. 

## References

[1] Witteveen T, Bezstarosti H, de Koning I, Nelissen E, Bloemenkamp KW, van Roosmalen J, van den Akker T (2017). Validating the WHO maternal near miss tool: comparing high- and low-resource settings. BMC Pregnancy Childbirth.

[2] Visi V, Akoijam BS (2021). A review of maternal near miss cases in selected hospitals in North-East India. Indian J Community Med.

[3] Alluvala SA, Aziz N, Tumkur A, Boorugu HK (2019). One-year follow-up of women with severe acute maternal morbidity (SAMM): a cohort study. J Obstet Gynaecol India.

[4] Chhabra P (2014). Maternal near miss: an indicator for maternal health and maternal care. Indian J Community Med.

[5] Kulkarni R, Kshirsagar H, Begum S, Patil A, Chauhan S (2021). Maternal near miss events in India. Indian J Med Res.

[6] Soma-Pillay P, Makin JD, Pattinson RC (2018). Quality of life 1 year after a maternal near-miss event. Int J Gynaecol Obstet.

[7] Upadhyay RP, Chowdhury R, Aslyeh Salehi (2017). Postpartum depression in India: a systematic review and meta-analysis. Bull World Health Organ.

[8] (2018). Niti Aayog: Maternal mortality ratio. http://niti.gov.in/content/maternal-mortality-ratio-mmr-100000-live-births.

[9] Callaghan WM, Grobman WA, Kilpatrick SJ, Main EK, D'Alton M (2014). Facility-based identification of women with severe maternal morbidity: it is time to start. Obstet Gynecol.

[10] (2018). National Health Mission: Maternal near-miss review operational guidelines. http://www.nrhmorissa.gov.in/writereaddata/Upload/Documents/Maternal_Near_Miss_Operational_Guidelines.pdf.

